# Analysis of influencing factors of economic burden and medical service utilization of diabetic patients in China

**DOI:** 10.1371/journal.pone.0239844

**Published:** 2020-10-30

**Authors:** Chen Chen, Jinglin Song, Xiaolan Xu, Leming Zhou, Yonghong Wang, Hong Chen

**Affiliations:** 1 Department of Public Economic System and Policy, School of Public Administration, Southwestern University of Finance and Economics, Chengdu, Sichuan, China; 2 Department of Statistics and Development Research, Chongqing Health Information Center, Chongqing, China; 3 Department of Clinical Laboratory, Chongqing Qianjiang Central Hospital, Chongqing, China; 4 Chongqing Institute of Translational Medicine, University of Chinese Academy of Sciences, Chongqing, China; Murdoch University, AUSTRALIA

## Abstract

**Background:**

This study analyzed factors that affect the financial burden and utilization of medical services of patients with diabetes in a city of China.

**Methods:**

We randomly sampled 10% of the information on the front page of diabetic inpatient medical records in the city from January 2014 to September 2019. Total cost of hospitalization, length of hospitalization and the number of hospitalization were analyzed. Descriptive statistical analysis and multiple linear regression analysis were adopted.

**Results:**

Understanding the current financial burden of diabetic patients and the use of medical services, the results show that the direct economic burden of diabetic patients per hospitalization was approximately 8,000 Yuan, and the indirect economic burden was approximately 2,000 Yuan. Age, medical payment methods, admission channels, and medical institution grades are all important factors affecting medical expenses and medical service utilization of diabetic patients. In addition, the inequality of medical service utilization of patients is increased due to different types of medical insurance.

**Conclusions:**

To reduce the economic burden on patients and society, governments should strengthen supervision, the advancement of diagnosis and treatment systems, the service conditions of primary medical institutions, the management of medical services, and the use of medical resources. To create a more impartial medical and health environment where the value of medical staff are truly reflected, financial investments should be attained to improve medical technologies and labor costs.

## Introduction

The disease spectrum of residents is changing with the increase in social living standards and modern life’s rhythm. Chronic noncommunicable diseases (NCD) such as diabetes, cardio- and cerebrovascular diseases, and cancer, are the main causes of disability and life expectancy reduction (accounting for greater than 80% of total deaths) [1, 2]. In the 1990s, there were approximately 100 million diabetics worldwide. By 2007, the number had grown rapidly to 246 million [3]. The most recent statistics of the International Diabetes Federation (IDF) indicate that the global diabetes prevalence rate was 8.3% in 2013, and the medical expenditure was 548 billion US dollars, accounting for 11% of global medical expenditures. Given that China’s total number of diabetic individuals has exceeded 98.4 million in 2013, surpassing India and ranking first around the world, the IDF estimates that the number will reach 143 million in China, while the related global medical expenditure will reach 627.3 billion US dollars by 2035 [4]. All above, diabetes has become an important risk factor for reduced human health, representing an increasing economic burden to human society [5].

Since the beginning of the last century, countries around the world have begun research on their burden of diabetes [6, 7]. The burden of disease refers to the loss and impact of disease, disability and premature death on life, health, and socioeconomics, including both epidemiological and economic burdens [8]. The economic burden of disease includes direct economic burden, indirect economic burden, and intangible burden. The disease epidemiological burden seriously harms human health, affects the quality of life, and causes significant physical and psychological pain to patients and their families. The economic burden caused directly or indirectly by the diseases represents a significant burden to many families on the other hand. Thus, poverty is increasingly serious due to the excessive economic burden of certain diseases in some regions of China.

In addition, the fairness and equality of medical service utilization should be an area of deeply concern given the increasing number of diabetics. The equity of medical service utilization can be categorized into horizontal equity and vertical equity. Horizontal equity is widely used in health economics and is defined as the notion that individuals with similar needs should obtain equal health care regardless of differences in income, region, and race. Horizontal equity is generally connected with age, self-health and other variables. Level inequality should apply to the use of medical services affected by nondemand variables, and individuals with the same needs receive different medical care [9]. Regarding the use of medical services, foreign scholars generally choose to measure the number of doctor appointments, emergency visits, and hospital stays within a certain period of time [10].

By analyzing the front page information of medical records among diabetics in a city of China, this research explores factors affecting the cost of diabetic inpatients and the utilization of medical resources and provides suggestions and a basis to improve the efficiency of the use of medical resources, increase the equality of medical resources utilization and formulate relevant strategies.

## Materials and methods

We randomly sampled 10% of the information on the front page of inpatient medical records of patients with primary diagnosis as "diabetes" in the city from January 2014 to September 2019 (the first three digit code of the 10th revision of the International Classification of Diseases (ICD-10) is E10 to E14), and this information was combined with the annual report information of medical institutions. The data include main diagnosis, other diagnoses, gender, age, length of stay, cost of stay, numbers of hospital visits, hospitalization expenses, age, gender, medical payment method, hospitalization way, occupation, marital status, and level of medical institution. These inpatient data were exclusively used to explore the cost of chronic diseases and the impact of medical service utilization. We signed a confidentiality agreement to guarantee that the above data will not be disclosed. All the data used in this article were obtained from the China Regional Health Information Platform, which collects administrative types of medical data. Importantly, personal information was completely inaccessible, and only objective variables can be analyzed under the restricted governance policy. The Regional National Health Committee of China approved all research procedures in this paper. With consent, a record was selected from the S1 File to make it easier for readers to understand the form of the data.

The economic burden of disease includes direct economic burden, indirect economic burden, and intangible economic burden. The direct economic burden refers to the economic resources directly consumed to prevent and cure diseases. We define the medical expense of hospitalization as a direct financial burden. The indirect economic burden is the economic loss to patients and society due to morbidity, disability and premature death. Indirect economic burden is calculated as the number of days of delay in the hospital multiplied by the average daily wage, in which the number of days of delay in hospital = (discharge date—admission date) + 1. The intangible economic burden is currently not calculated given the lack of an accurate measurement method.

The Charlson Comorbidity Index (CCI) is a scoring system based on the number and severity of illnesses in patients and involves weighting factors (the rating scale is shown in S1 Table) [11]. We sorted and assigned the patient’s comorbidities data. If the patient has no comorbidities, the CCI score is 0. If the patient only has 1 comorbidity with a weight of 1, the CCI score is 1. If a patient has two comorbidities with a weight of 1 or has one comorbidity with a weight of 2, the CCI score is 2. If a patient has comorbidities with a weight sums ≥ 3, then the CCI score is 3.

The corresponding statistical analysis was performed using stata15 software. Descriptive statistical analysis and multiple linear regression analysis were adopted. In the multiple linear regression analysis, the logarithm change method was used to transform the variables that do not exhibit a normal distribution, and the year fixed effect was also applied. The logarithmic change method is to determine the logarithm of the variables to be explained, and then insert multiple linear model for regression. There are two reasons why we use the logarithmic variation method. Firstly, this logarithm is used to improve the goodness of fit of regression model. Secondly, we want to interpret the regression coefficient as semi elastic (i.e., percentage change). Dummy variables were used to classify variables, and continuous variables were directly included in the analysis. Regarding dependent variables, we chose the total cost of hospitalization to measure the direct economic burden of diabetic individuals, the length of hospitalization and the number of hospitalizations to measure the utilization level of medical services. The independent variables included patient age, gender, medical payment method, hospitalization way, diabetes type, CCI score, numbers of hospital visits, hospitalization days, occupation, marital status, and hospital level (Table 1). Among them, the hospital level in China is relatively special, which is unified by the health administrative department according to the comprehensive level of hospital functions, tasks, facilities, technical construction, medical service quality and scientific management. Among them, primary hospitals include rural township hospitals and urban street hospitals; secondary hospitals include provincial and urban general hospitals, county-level hospitals and provincial urban hospitals, as well as staff hospitals with considerable scale in industrial and mining enterprises and institutions; and tertiary hospitals include national, provincial and municipal hospitals directly under the central government and affiliated hospitals of medical colleges and universities.

**Table 1:** Variable and assignment description.

Variables name	Variables type	Assignment
**Method of Medical payment**	Dummy variable	0 = NRCMS 1 = UEBMI; 2 = URBMI;3 = Others
**Pathway of Hospitalization**	Dummy variable	0 = Emergency; 1 = Outpatient; 2 = Others
**Type of diabetes**	Dummy variable	0 = Type 1; 1 = Type 2; 2 = Others
**Gender**	Dummy variable	0 = Female;1 = Male
**CCI**	Dummy variable	0 = CCI0;1 = CCI1;2 = CCI2;3 = CCI3
**Marriage status**	Dummy variable	0 = Single; 1 = Married; 2 = Widowed; 3 = Divorced; 4 = Others
**Occupation**	Dummy variable	0 = Unemployment;1 = Farmer; 2 = Retire;3 = Self-employment; 4 = Employment; 5 = Others
**Hospital level**	Dummy variable	0 = Primary;1 = Secondary; 2 = Tertiary; 3 = Others
**Length of stay**	Continuous variable	
**Number of hospitalization**	Continuous variable	
**Age**	Continuous variable	

NRCMS, the new rural cooperative medical insurance; UEBMI, the basic medical insurance for urban employees; URBMI, the basic medical insurance for urban residents. CCI, the Charlson Comorbidity Index.

The Charlson Comorbidity Index (CCI) is a scoring system based on the number and severity of illnesses in patients and involves weighting factors. Their characteristics are shown in Table 2 [11]. We sorted and assigned the patient’s comorbidities data. If the patient has no comorbidities, the CCI score is 0; If the patient only has 1 comorbidity with weights of 1, the CCI score is 1; If a patient has two comorbidities with weights of 1 or has one comorbidity with weights of 2, the CCI score is 2; If a patient has comorbidities with a sum of weights ≥ 3, then the CCI score is 3.

**Table 2:** Basic characteristics of patients.

Characteristics		Frequency	Percentage
**Gender**	**Male**	14096	49.36%
	**Female**	14464	50.64%
**Age group**	**<18**	295	1.03%
	**18–44**	2,647	9.27%
	**45–59**	8,640	30.25%
	**60–74**	11,804	41.33%
	**75–89**	4,993	17.48%
	**>89**	181	0.63%
**Medical payment method**	**UEBMI**	12,255	42.91%
	**URBMI**	6,515	22.81%
	**NRCMS**	2,697	9.44%
	**Others**	7,093	24.84%
**Hospitalization Pathway**	**Emergency**	6,264	21.93%
	**Outpatient**	17,903	62.69%
	**Others**	4,393	15.38%
**Occupation**	**Unemployment**	1,202	4.21%
	**Farmer**	6,660	23.32%
	**Retire**	4,706	16.48%
	**Self-employment**	762	2.67%
	**Employment**	3,362	11.77%
	**Others**	11,868	41.55%
**Marriage status**	**Single**	1,211	4.24%
	**Married**	25,178	88.16%
	**Widowed**	987	3.46%
	**Divorced**	390	1.37%
	**Others**	794	2.78%
**Type of diabetes**	**Type 1**	632	2.21%
	**Type 2**	26,123	91.47%
	**Others**	1,805	6.32%
**CCI**	**0**	5951	20.84%
	**1**	2072	7.25%
	**2**	2109	7.38%
	**3**	18428	64.52%
**Length of stay**	**<5**	6,820	23.88%
	**5–10**	13,022	45.60%
	**11–15**	5,588	19.57%
	**>15**	3,130	10.96%
**Hospital level**	**Primary hospitals**	482	1.69%
	**Secondary hospitals**	12871	45.07%
	**Tertiary hospitals**	13425	47.01%
	**Others**	1782	6.24%

NRCMS, the new rural cooperative medical insurance; UEBMI, the basic medical insurance for urban employees; URBMI, the basic medical insurance for urban residents. CCI, the Charlson Comorbidity Index.

## Results

### Analysis of basic characteristics

**Fig 1:**
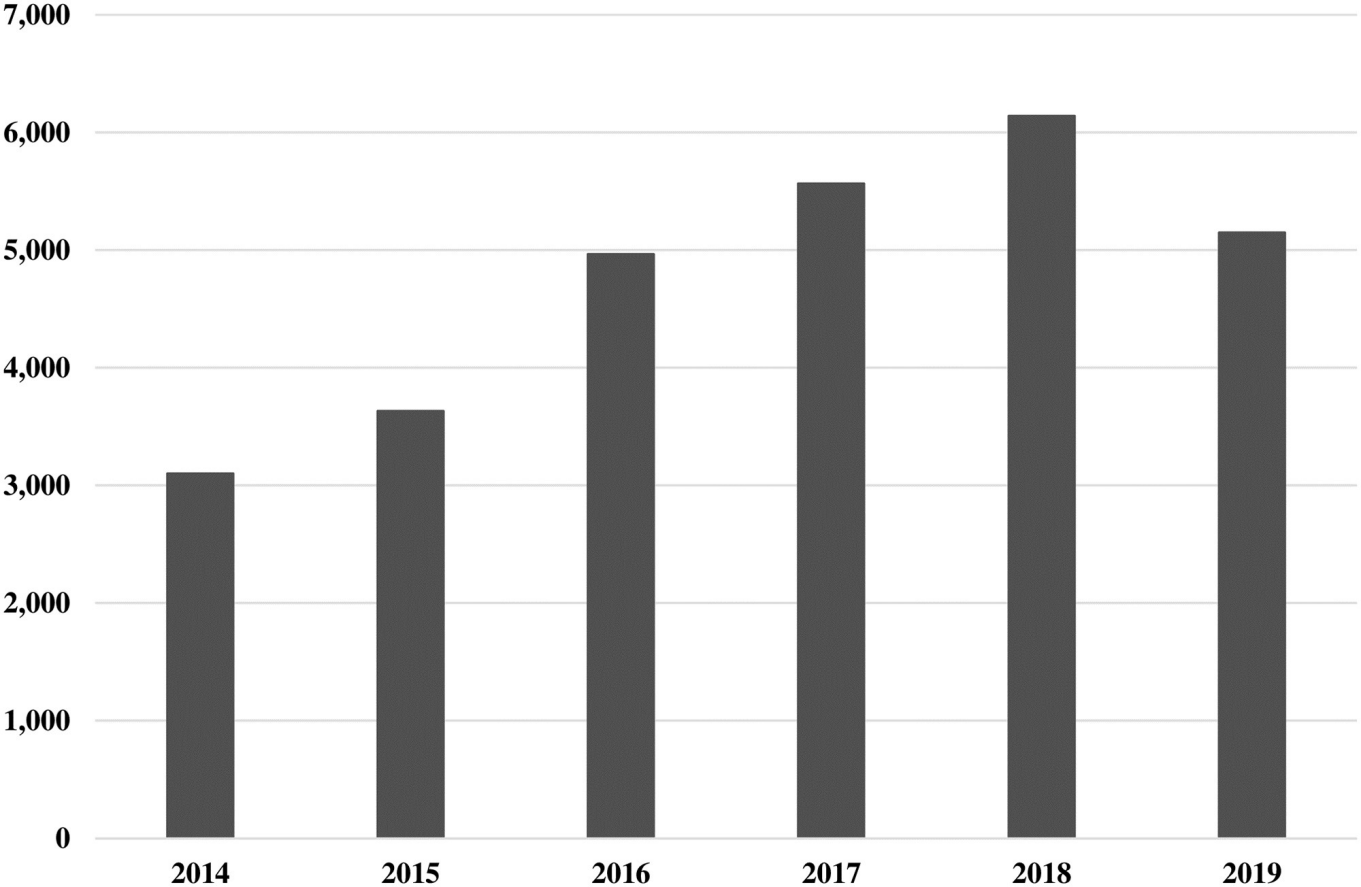
Number of diabetic patients in data sample between January 2014 and September 2019.

Our total data sample includes 1430967 inpatients from January 2014 to September 2019, accounting for 10% of the total number of discharged patients in the city. The data used in this study are obtained by screening the main diagnosis of "diabetes" (the first three codes of ICD-10 are E10 to E14). As shown in Fig 1, 28560 patients visits covering 312 hospitals were included. Based on the descriptive statistics in Table 2, the percentage of female cases was 50.64%, which was increased compared with male cases. Approximately 90% patients were over 45 years old, and the average age was 61 years old. In total, 42.91% of medical expenses were paid by the basic medical insurance for urban employees (UEBMI), while the basic medical insurance for urban residents (URBMI) and the new rural cooperative medical insurance (NRCMS) account for approximately 22% and 9%, respectively. Among the admission methods, 21.93% of cases were admitted to the emergency department, whereas 62.69% of cases were admitted to the outpatient department. Regarding occupations, 23.32% of the patients were farmers. Married cases accounted for 88.16%. Type 2 diabetes was the main type, representing 91.47%. 20.84%, 7.25%, 7.38% and 64.52% of cases were assigned CCI scores of 0, 1, 2 and 3, respectively. The average hospital stay was approximately 9 days, and the length of stay was 5–10 days in 13022 cases, representing 45.60%. Most of the patients chose to go to secondary and tertiary medical institutions, accounting for approximately 92%.

### Analysis of the composition of average hospitalization expenses

The total cost of hospitalization is composed of Western medicine expenses, examination expenses, treatment expenses, service expenses, material expenses, blood expenses, traditional Chinese medicine treatment expenses, traditional Chinese medicine expenses and other expenses. Among them, the highest proportion of average costs was Western medicine and examination at 2745.47 Yuan (32.87%) and 2723.87 Yuan (32.61%) respectively. The average treatment fee per time was 386.79 Yuan, accounting for 4.63%. The average service fee per time was 1241.26 Yuan, (14.66% of the total expenses). The average material fee per time was 348.76, accounting for 4.18%. The average blood fee per time was 13.83 Yuan, accounting for 0.17%. The average treatment fee per time was 18.15 Yuan (0.22%). The average Chinese medicine fee per time and the average of additional fees per time were 297.98 and 608.27 Yuan, respectively, accounting for 3.57% and 7.11% of their total respective expenses. See Table 3 for details.

**Table 3:** Composition of hospitalization expenses (Yuan).

Characteristics	Mean	SD	Min	Max	Percentage
**Western medicine Expenditure**	2745.473	3730.469	13.96	213958.3	32.87%
**Check Expenditure**	2723.868	2610.906	20.41	122936.57	32.61%
**Treatment Expenditure**	386.793	1243.653	0	104528.49	4.63%
**Serve Expenditure**	1241.258	2764.144	8.65	107696.48	14.66%
**Material Expenditure**	348.755	1510.1	0	87945.297	4.18%
**Blood Expenditure**	13.825	180.918	0	8382.410	0.17%
**Other Expenditure**	608.267	2880.245	0	219614.2	7.11%
**TCM treatment Expenditure**	18.145	277.278	0	31610.16	0.22%
**TCM Expenditure**	297.982	660.977	0	17323.428	3.57%
**Total Expenditure**	8353.368	9782.142	46.82	574079.25	100%

TCM, traditional Chinese medicine. SD, standard deviation.

### Analysis of direct economic burden and utilization of medical services

As shown in Table 4, the average hospitalization cost of diabetic patients was 8353.368 Yuan, so the direct economic burden of diabetes was approximately 8000 Yuan. Specific differences are noted among patients with different types of insurance payments. The direct economic burden of using the basic medical insurance for urban workers was approximately 9900 Yuan. The direct economic burden of using the basic medical insurance of urban residents was approximately 7300 Yuan and that of using the new rural cooperative medical insurance was approximately 7000 Yuan. Regarding indirect economic burden the average number of days off work of diabetic patients was 10.882 days, and the average daily wage of the city from 2014 to 2019 was approximately 200 Yuan. Thus, the indirect economic burden of diabetic patients was approximately 2000 Yuan. The indirect economic burden of patients was approximately 2470 Yuan, 1920 Yuan and 1860 Yuan based on the time lost from work of UEBMI (12.345 days), URBMI (9.596 days) and NRCMS (9.324 days), respectively. Focusing on medical service utilization, the average length of stay for diabetes in this city was 9.882 days, and the number of visits is 1.727. From the perspective of different payment methods of medical expenses, regardless of the total medical expenses, hospitalization days, or numbers of hospital visits, the three characteristic values for patients who use UEBMI rank the highest followed by people using URBMI and NRCMS.

**Table 4:** Dependent variable characteristics.

Characteristics	Mean	SD
**Total Expenditure**		
**NRCMS**	7005.569	11936.39
**URBMI**	7365.604	6453.112
**UEBMI**	9930.333	11701.43
**Total**	8353.368	9782.142
**Length of stay**		
**NRCMS**	8.324	5.802
**URBMI**	8.596	6.307
**UEBMI**	11.345	13.392
**Total**	9.882	10.582
**Numbers of hospital visits**		
**NRCMS**	1.189	0.805
**URBMI**	1.351	1.314
**UEBMI**	1.993	4.570
**Total**	1.727	3.372

NRCMS, the new rural cooperative medical insurance; UEBMI, the basic medical insurance for urban employees; URBMI, the basic medical insurance for urban residents. SD, standard deviation.

### Multiple regression analysis of the influencing factors of the average total cost of hospitalization

The results indicate that the main influencing factors of the total cost of inpatients were length of stay, methods of medical payment, pathway of hospitalization, marriage status, CCI score and grade of medical institution (P < 0.05). The coefficient of method of medical payment shows that after controlling the patient’s personal characteristics and the level of medical institutions, the medical expenses paid by the urban employee medical insurance were increased by 19.6% compared with those paid by the new rural cooperative medical system. The medical expenses of patients who use medical insurance for urban residents were increased by 6.8% compared with those paid by the new rural cooperative medical system. The medical expenses of patients with other payment methods were increased by 9% compared with the new rural cooperative medical system. When the length of stay increased one day, total medical expenses increased by 3.2%. In addition, the emergency patients paid more on average hospitalization cost. Meanwhile, type 2 diabetic patients spent more than type 1 diabetics. The higher the CCI index, the higher the average cost of patients. The average cost of patients visiting tertiary or secondary medical institutions was increased compared with patients visiting primary medical institutions (Table 5).

**Table 5:** Multiple regression analysis of the average total cost of hospitalization.

Variable	Coefficient	S.E	t-Statistics	P Value	Lower 95%CI	Upper 95%CI
**Age**	-0.001	0.002	-0.660	0.507	-0.005	0.002
**Age square**	0.000	0.000	2.87	0.004	0.000	0.000
**Gender** **(Male = 1 Female = 0)**	0.015	0.010	1.550	0.120	-0.004	0.035
**Length of stay**	0.032	0.000	68.460	<0.001	0.031	0.033
**Number of hospital visits**	0.002	0.001	1.330	0.184	-0.001	0.005
**Method of medical payment**						
**UEBMI**	0.196	0.019	10.30	0.000	0.159	0.234
**URBMI**	0.068	0.019	3.550	0.000	0.031	0.106
**Others**	0.090	0.020	4.570	0.000	-0.126	-0.078
**Pathway of Hospitalization**						
**Outpatient**	-0.102	0.012	-8.360	0.000	-0.126	-0.078
**Others**	-0.131	0.016	-8.090	0.000	-0.162	-0.099
**Type of diabetes**						
**Type 2**	0.046	0.037	1.230	0.217	-0.027	0.118
**Others**	-0.019	0.041	-0.460	0.648	-0.099	0.062
**CCI**						
**CCI1**	0.265	0.023	11.690	<0.001	0.221	0.310
**CCI2**	0.275	0.023	12.040	<0.001	0.230	0.320
**CCI3**	0.496	0.017	28.960	<0.001	0.463	0.530
**Occupation**						
**Farmer**	-0.016	0.025	-0.650	0.518	-0.065	0.033
**Retire**	-0.047	0.026	-1.830	0.068	-0.097	0.003
**Self-employment**	-0.018	0.027	-0.660	0.506	-0.071	0.035
**Employment**	-0.019	0.038	-0.490	0.621	-0.093	0.056
**Others**	-0.142	0.028	-5.03	0.000	-0.198	-0.087
**Marriage status**						
**Married**	-0.144	0.027	-5.360	0.000	-0.198	-0.092
**Widowed**	-0.143	0.037	-3.880	0.000	-0.216	-0.071
**Divorced**	-0.235	0.049	-4.860	0.000	-0.331	-0.141
**Others**	-0.254	0.040	-6.420	0.000	-0.331	-0.176
**Hospital level**						
**Secondary hospitals**	0.365	0.038	9.610	<0.001	0.290	0.439
**Tertiary hospitals**	0.722	0.038	18.880	<0.001	0.647	0.797
**Others**	0.367	0.042	8.830	<0.001	0.286	0.449
**cons**	6.968	0.058	120.390	<0.001	6.854	7.081

S.E, standard error. 95%CI, 95% confidence interval. UEBMI, the basic medical insurance for urban employees; URBMI, the basic medical insurance for urban residents. CCI, the Charlson Comorbidity Index.

### Multiple regression analysis of the influencing factors of the average length of stay

According to Table 6, we found that gender, number of hospitalizations, medical payment method, pathway of hospitalization, type of diabetes, marriage status, CCI score and grade of medical institutions were the main factors affecting the average length of stay of diabetic patients (P < 0.05).

**Table 6:** Multiple regression analysis of the average length of stay.

Variable	Coefficient	S.E	t-Statistics	P Value	Lower 95%CI	Upper 95%CI
**Age**	-0.001	0.001	-0.680	0.499	-0.004	0.002
**Age square**	0.000	0.000	3.650	0.000	0.000	0.000
**Gender** **(Male = 1 Female = 0)**	0.024	0.008	2.990	0.003	0.008	0.040
**Number of hospital visits**	0.016	0.001	13.780	0.000	0.014	0.018
**Method of medical payment**						
**UEBMI**	0.183	0.016	11.660	0.000	0.152	0.214
**URBMI**	0.051	0.016	3.190	0.001	0.019	0.082
**Others**	0.056	0.016	3.450	0.001	0.024	0.088
**Pathway of Hospitalization**						
**Outpatient**	-0.035	0.010	-3.530	0.000	-0.055	-0.016
**Others**	-0.003	0.013	-0.230	0.817	-0.029	0.023
**Type of diabetes**						
**Type 2**	0.070	0.030	2.290	0.022	0.010	0.129
**Others**	0.123	0.034	3.620	0.000	0.056	0.189
**CCI**						
**CCI1**	0.076	0.019	4.050	0.000	0.039	0.113
**CCI2**	0.054	0.019	2.850	0.004	0.017	0.090
**CCI3**	0.208	0.014	14.750	0.000	0.181	0.236
**Occupation**						
**Farmer**	0.027	0.020	1.340	0.181	-0.013	0.067
**Retire**	-0.047	0.021	-2.240	0.025	-0.088	-0.006
**Self-employment**	0.020	0.022	0.890	0.375	-0.024	0.064
**Employment**	0.040	0.031	1.290	0.197	-0.021	0.102
**Others**	0.043	0.023	1.840	0.065	-0.003	0.088
**Marriage status**						
**Married**	-0.129	0.022	-5.790	0.000	-0.172	-0.085
**Widowed**	-0.082	0.030	-2.680	0.007	-0.141	-0.022
**Divorced**	0.050	0.040	1.260	0.207	-0.028	0.128
**Others**	-0.111	0.033	-3.420	0.001	-0.175	-0.047
**Hospital level**						
**Secondary hospitals**	0.034	0.031	1.080	0.278	-0.027	0.096
**Tertiary hospitals**	0.142	0.032	4.480	0.000	0.080	0.203
**Others**	0.104	0.034	3.030	0.002	0.037	0.172
**cons**	1.747	0.058	30.090	0.000	1.633	1.860

S.E, standard error. 95%CI, 95% confidence interval. UEBMI, the basic medical insurance for urban employees; URBMI, the basic medical insurance for urban residents. CCI, the Charlson Comorbidity Index.

After controlling for the patient’s personal characteristics and hospital level, the length of stay of patients paid by urban employee medical insurance was 18.3% longer compared with that of patients paid by the new rural cooperative medical system. The hospitalization days of general outpatient patients were 3.5% shorter than those of emergency patients. Type 2 diabetic patients stayed longer than type 1 diabetic patients. The results showed that the length of stay of patients with type 2 diabetes mellitus was 7% longer than that of patients with type 1 diabetes mellitus. The length of stay of patients who went to a second-level hospital was 3.4% longer than that of patients who went to a first-level hospital; the hospitalization days of patients who went to a third-level hospital were 14.2% longer than those who went to a first-level hospital. In addition, the length of stay of male patients was longer than that of female patients. The higher the CCI index, the longer the length of stay.

### Multiple regression analysis of influencing factors of numbers of hospital visits

Through multiple regression analysis of numbers of hospital visits, we found that the influencing factors of numbers of hospital visits of diabetic patients were age, hospitalization days, medical payment method, marriage and types of medical institutions (P<0.05). It should be noted that after controlling other variables, the hospitalization days of patients paid by urban employee medical insurance were increased by 11.5% compared with those paid by new rural cooperative medical system. No significant difference were noted between the hospitalization days paid by urban employee medical insurance and the number of hospital visits of patients paid by new rural cooperative medical treatment (P < 0.05). The number of hospital visits of standard inpatients was increased by 6.1% compared with that of emergency patients. The number of hospital visits for type 2 diabetes patients was 16.2% reduced compared with those for type 1 diabetic patients. The number of hospital visits of self-employment patients was increased by 19% compared with that of unemployed patients. No significant difference in the number of hospitalizations was noted between patients who went to tertiary and secondary medical institutions compared with patients who went to primary medical institutions (see Table 7).

**Table 7:** Multiple regression analysis of numbers of hospital visits.

Variable	Coefficient	S.E	t-Statistics	P Value	Lower 95%CI	Upper 95%CI
**Age**	-0.005	0.001	-3.810	0.000	-0.007	-0.002
**Age square**	0.000	0.000	8.10	0.000	0.000	0.000
**Gender** **(Male = 1 Female = 0)**	-0.015	0.007	-2.10	0.036	-0.029	-0.001
**Length of stay**	0.008	0.000	23.820	0.000	0.007	0.008
**Method of medical payment**						
**UEBMI**	0.115	0.014	8.450	0.000	0.088	0.141
**URBMI**	0.024	0.014	1.730	0.084	-0.003	0.050
**Others**	0.179	0.014	12.80	0.000	0.151	0.206
**Pathway of Hospitalization**						
**Outpatient**	0.061	0.009	6.990	0.000	0.044	0.078
**Others**	0.038	0.011	3.290	0.001	0.015	0.060
**Type of diabetes**						
**Type 2**	-0.162	0.026	-6.160	0.000	-0.213	-0.110
**Others**	-0.164	0.029	-5.620	0.000	-0.222	-0.107
**CCI**						
**CCI1**	0.007	0.016	0.460	0.645	-0.024	0.039
**CCI2**	-0.016	0.016	-0.960	0.339	-0.047	0.016
**CCI3**	0.028	0.012	2.310	0.021	0.004	0.052
**Occupation**						
**Farmer**	0.056	0.018	3.160	0.002	0.021	0.090
**Retire**	-0.013	0.018	-0.710	0.475	-0.049	0.023
**Self-employment**	0.190	0.019	9.840	0.000	0.152	0.228
**Employment**	0.022	0.027	0.810	0.417	-0.031	0.075
**Others**	0.026	0.020	1.270	0.203	-0.014	0.065
**Marriage status**						
**Married**	-0.001	0.019	-0.030	0.974	-0.038	0.037
**Widowed**	0.129	0.026	4.910	0.000	0.078	0.181
**Divorced**	0.037	0.035	1.080	0.282	-0.031	0.105
**Others**	-0.125	0.028	-4.450	0.000	-0.180	-0.070
**Hospital level**						
**Secondary hospitals**	-0.037	0.027	-1.380	0.167	-0.091	0.016
**Tertiary hospitals**	-0.007	0.027	-0.240	0.809	-0.060	0.047
**Others**	-0.078	0.030	-2.630	0.008	-0.137	-0.020
**cons**	-0.106	0.050	-2.120	0.034	-0.205	-0.008

S.E, standard error. 95%CI, 95% confidence interval. UEBMI, the basic medical insurance for urban employees; URBMI, the basic medical insurance for urban residents. CCI, the Charlson Comorbidity Index.

## Discussion

Diabetes, one of the major chronic diseases in the world, is difficult to cure and exhibits a long disease course. The health and daily lives of patients are seriously affected by the enormous medical burden. The results of this study show that the direct economic burden of diabetic patients in the city was approximately 8000 Yuan, whereas the indirect economic burden was approximately 2000 Yuan. Based on the income level of the region, and the fact that approximately 90% of patients over 45 years old and most were elderly, the majority of these individuals would bear a huge economic burden given the limited income. In the composition of hospitalization expenses, Western medicine expenses and examination expenses accounted for approximately 70% of the total. Based on this proportion, unreasonable problems in the composition of hospitalization expenses emerge [12–14]. By using more drugs and examinations, hospitals could maximize their profits, thus ensuring hospital survival. Given the high proportion of drug costs among all the medical expenses of diabetic patients, drugs remain the primary method of control, and diet or other nondrug treatment methods are less often used. Based on the above analysis, we suggest that the government should increase the price of diagnosis, treatment, services and other items and supervise medical expenses to improve the efficiency of medical resource utilization and reduce the economic burden of patients.

Research shows that primary medical institutions are the most important platform for chronic disease managements, and community health service centers can play a better role in diabetes prevention and monitoring [15]. Our results show that less than 10% diabetic patients in the sample go to primary medical institutions although they would more and stay longer in secondary and tertiary medical institutions. We should speed up hierarchical diagnosis and treatment and make use of primary prevention by performing blood glucose tests and physical examinations in residents regularly. We will continue activities to improve public awareness of diabetes, early detection and treatment to reduce the common economic burden.

From the results of multiple regressions, it can be seen that the length of stay in hospital is also an important factor affecting medical expenses. The hospitalization cost increased as the cost of medicine, examination, bed and service increased during the prolonged hospitalization, which is consistent with other research results [15–17]. Therefore, the hospital should make full use of the existing resources to regulate the behavior of doctors, enhancing the working efficiency of all departments. By reducing inefficient hospitalization time utilization, bed turnover will be increased, and the economic burden of patients will be reduced. In addition, age and the number and severity of comorbidities also affect medical costs and the length of stay of patients, which is consistent with the results of other studies [18, 19]. In addition, patients may spend more on medical expenses and stay longer in hospitals as age and comorbidities increase. Therefore, given the increase in the ageing population, we should make relevant knowledge universal and improve the medical security system to enable diabetic patients to obtain more rapid and standardized treatment to decrease complications and comorbidities.

In addition, we should increase attention regarding fairness of medical resource utilization on the background of the universal coverage of China’s basic medical insurance. Our results demonstrate that medical expenses and medical resources utilization of diabetic patients with medical insurance for urban residents are significantly increased compared with patients with other types of insurance [20, 21]. As the insurance level and reimbursement ratio of basic medical insurance for urban employees are better than other types of basic medical insurance, the excessive consumption of insured persons and the induced demand from medical institutions continually emerge. All of these situations further weaken fair medical resource utilization. Therefore, governments should improve medical service management and the utilization of medical resources, strengthen the supervision of drug prices and inspection items, and curb unnecessary prescriptions and unreasonable inspections [22]. Investments in medical service providers, such as improving the benefit of medical personnel and the service conditions of primary medical institutions, should be prioritized to create a more equitable medical and health environment.

## Conclusion

The results of this study show that the direct economic burden of diabetes patients was approximately 8000 Yuan, and the indirect economic burden was approximately 2000 Yuan. Age, the method of medical payment, the method of admission and the level of medical institutions are all important factors that affect medical expenses and the utilization of medical services of diabetic patients. In addition, different types of health insurance may exacerbate inequalities in health service utilization due to differences in thresholds and reimbursement rates (S2 Table). Therefore, the government should strengthen the supervision of cost composition and make steady progress in the tiered diagnosis and treatment model. We should make better use of primary medical institutions and improve service conditions, medical service management and medical resources utilization with the aim to reduce the economic burden of patients and society. Finally, we should increase financial input, improve medical technology and labor costs that truly reflect medical personnel value, and strengthen communication and coordination at all levels to create a more equitable medical and health environment.

## Supporting information

S1 Table CCI score of Chalson complication index. (DOCX)

S2 Table Thresholds for reimbursement (yuan) and reimbursement ratio of medical expenses (%). (DOCX)

S1 File Data sample. (DTA)
