# Supplementary material for: Analysis of influencing factors of economic burden and medical service utilization of diabetic patients in China
Source: PLoS One. 2020 Oct 30;15(10):e0239844. doi: 10.1371/journal.pone.0239844 (PMC7598469; doi:10.1371/journal.pone.0239844)
Supplement: S1 Table — (DOCX) [file pone.0239844.s001.docx]

**S1 Table. CCI score of Chalson complication index.**

| Condition | Weights | ICD-10-AM |
| --- | --- | --- |
| Acute myocardial infarction | 1 | I21, I22, I252 |
| Congestive heart failure | 1 | I50 |
| Peripheral vascular disease | 1 | I71, I790, I739, R02, Z958, Z959 |
| Cerebral vascular accident | 1 | I60, I61, I62, I63, I65, I66,G450, G451, G452, G458, G459, G46,I64, G454, I670, I671, I672, I674, I675, I676, I677 I678, I679,I681, I682, I688, I69 |
| Dementia | 1 | F00, F01, F02, F051 |
| Pulmonary disease | 1 | J40, J41, J42, J44, J43, J45, J46, J47, J67, J44, J60, J61,J62, J63, J66, J64, J65 |
| Connective tissue disorder | 1 | M32, M34, M332, M053, M058, M059, M060, M063, M069,M050, M052, M051, M353 |
| Peptic ulcer | 1 | K25, K26, K27, K28 |
| Liver disease | 1 | K702, K703, K73, K717, K740, K742, K746, K743, K744, K745 |
| Diabetes | 1 | E109, E119, E139, E149, E101, E111, E131, E141, E105, E115, E135, E145 |
| Diabetes complications | 2 | E102, E112, E132, E142 E103, E113, E133, E143 E104, E114, E134, E144 |
| Paraplegia | 2 | G81 G041, G820, G821, G822 |
| Renal disease | 2 | N03, N052, N053, N054, N055, N056, N072, N073, N074, N01,N18, N19, N25 |
| Cancer | 2 | C0, C1, C2, C3, C40, C41, C43, C45, C46, C47, C48, C49, C5,C6, C70, C71, C72, C73, C74, C75, C76, C80, C81, C82, C83,C84, C85, C883, C887, C889, C900, C901, C91, C92,C93, C940, C941, C942, C943, C9451, C947,C95, C96 |
| Metastatic cancer | 3 | C77, C78, C79, C80 |
| Severe liver disease | 3 | K729, K766, K767, K721 |
| HIV | 6 | B20, B21, B22, B23, B24 |
