# Supplementary material for: Analysis of influencing factors of economic burden and medical service utilization of diabetic patients in China
Source: PLoS One. 2020 Oct 30;15(10):e0239844. doi: 10.1371/journal.pone.0239844 (PMC7598469; doi:10.1371/journal.pone.0239844)
Supplement: S2 Table — (DOCX) [file pone.0239844.s002.docx]

**S2 Table. Thresholds for reimbursement（yuan）and reimbursement ratio of medical expenses（%）.**

|  | UEBMI | | | URBMI | | |
| --- | --- | --- | --- | --- | --- | --- |
|  | Thresholds for reimbursement | Employment | Retire | Thresholds for reimbursement | Low-grade | High-grade |
| Tertiary hospitals | 880 | 85 | 95 | 800 | 40 | 45 |
| Secondary hospitals | 440 | 87 | 95 | 300 | 60 | 65 |
| Primary hospitals | 200 | 90 | 95 | 100 | 80 | 85 |
